# Factors influencing home dialysis choice in Scandinavia: a cross-sectional study

**DOI:** 10.1186/s12882-026-04972-4

**Published:** 2026-04-14

**Authors:** Astrid Torbjørnsen, Beate Nygaard-Andersen, Kiana Kiani, Ann-Chatrin Linqvist Leonardsen, Axel Wolf, Are Hugo Pripp, Peter Forde Hougaard, Jeanette Finderup

**Affiliations:** 1 Department of Nursing and Health Promotion, Oslo Metropolitan University – OsloMet, Pilestredet 32, Oslo, 0130 Norway; 2 Research Department, Lovisenberg Diaconal Hospital, Oslo, Norway; 3 Centre for Person-Centred Care (GPCC), University of Gothenburg, Gothenburg, Sweden; 4 Faculty of Health, Welfare and Organization, Østfold University College, Halden, Norway; 5 Department of Research, Østfold Hospital Trust, Grålum, Norway; 6 Department of Renal Medicine, Aarhus University Hospital, Aarhus, Denmark; 7 Department of Clinical Medicine, Aarhus University, Aarhus, Denmark

**Keywords:** Home dialysis, Mixed-method design, Cross-sectional study, Scandinavia

## Abstract

**Background:**

Home dialysis provides several advantages over in-center hemodialysis, yet its uptake remains limited. Understanding how patients and kidney professionals perceive home dialysis is key to improving access and support. The aim of this study was to identify factors influencing the choice of home dialysis and to examine potential differences in perceptions between patients with kidney failure and kidney professionals across countries.

**Methods:**

A cross-sectional study using mixed-methods explanatory sequential design was conducted. A digital survey, developed through eight workshops (2021–2023) with patients and kidney professionals, included both closed- and open-ended questions. The survey was distributed to patients with kidney failure and kidney professionals in Scandinavia. Quantitative data were analyzed using logistic regression adjusted for gender, age, and country, while qualitative responses underwent content analysis.

**Results:**

The survey received responses from 269 patients and 185 professionals. Patients were predominantly male (57.3%, mean age 61.3), while professionals were mainly female (85.6%, mean age 49.4). Prior patient experience included PD in 179 (35.8%) and HHD in 66 (13.2%). Independence was identified as the top reason for choosing home dialysis, though many found the decision challenging. Patients cited barriers such as unclear reimbursement (OR = 0.29), limited technology (OR = 0.44), and poor communication tools (OR = 0.18). Professionals highlighted patient reluctance (OR = 7.29), relative involvement (OR = 7.54), social isolation (OR = 7.18), and unsuitable home environments (OR = 3.64). Qualitative responses highlighted the complexity of modality choice and illustrated differing perspectives between patients and professionals.

**Conclusions:**

Patients and professionals perceive distinct barriers to home dialysis. Tailored interventions addressing these differences are essential to improve the uptake of home dialysis in Scandinavia.

**Trial registration:**

NA.

**Supplementary Information:**

The online version contains supplementary material available at 10.1186/s12882-026-04972-4.

## Background

Studies indicate that home dialysis, encompassing peritoneal dialysis (PD) and home hemodialysis (HHD), offers important advantages over in-center hemodialysis, including improved quality of life, increased autonomy, and reduced healthcare costs [1, 2]. Despite these benefits, the uptake of home dialysis remains limited, even among patients who are clinically eligible [3, 4]. According to Lundström et al. [5], the prevalence and incidence of home dialysis vary significantly across Europe, with countries offering structured pre-dialysis education and supportive national guidelines generally reporting higher uptake. Denmark, Sweden, and Norway are among the top countries in Europe in terms of both prevalent and incident home dialysis patients [5]. Yet even in these settings, home dialysis remains underutilized. Recent registry data indicate that approximately 20–30% of dialysis treatments in the Scandinavian countries are delivered at home, although the exact proportions vary between national healthcare systems and regions. Despite relatively high uptake compared with many other European countries, the proportion of patients treated with home dialysis remains below the level considered achievable for clinically eligible patients. It has been estimated that up to 50% of patients could benefit from home dialysis options, suggesting that current uptake falls well below the potential [5].

A growing body of literature highlights multiple barriers that affect dialysis modality choice. These barriers exist at the level of the patient, the kidney professional, and the healthcare system: Patients and caregivers often perceive home dialysis as a way to maintain independence and daily routines, but they also report concerns about isolation, treatment burden, and a lack of ongoing support [2, 6, 7]. On the provider side, limited experience with home dialysis and ambivalence toward recommending it can discourage its uptake [8].

Several factors in the pre-dialysis phase are particularly influential. According to Bonenkamp et al. [9], both patients and kidney professionals identify significant obstacles to choosing home dialysis, including insufficient information and education, logistical and financial barriers, treatment complexity, lack of infrastructure, and inadequate resources for patient support. Moreover, patients’ perceptions of their illness, as well as kidney professionals’ attitudes toward different dialysis options, can have a substantial impact on treatment decisions [10, 11]. Comprehensive pre-dialysis education, access to peer experiences, and consistent messaging from the care team have been suggested as critical components of informed, person-centered decision-making [1, 12].

This points to the need for a more person-centered, supportive approach to dialysis modality choice, that involves patients actively in decisions and better addresses the practical and psychological barriers to home treatment [13].

### Aim

The aim of the current study was to examine the perceptions of patients with kidney failure and kidney professionals regarding factors influencing the choice of home dialysis, as well as potential differences between patients and kidney professionals, and across the Scandinavian countries.

## Methods

### Design

The study had a cross-sectional design with a mixed-methods explanatory sequential design, utilizing a survey combining closed-ended questions, each of which was followed by an open-ended commentary box. This study was reported according to the EQUATOR Consensus-Based Checklist for Reporting of Survey Studies [14].

### Setting and sample

The study was conducted transnationally, with inclusion criteria including all adult (≥ 18 years old) patients with kidney failure who have experience with dialysis (present or past), encompassing all kidney professionals with experience in PD, HD, and nephrology clinics, as well as kidney professionals working in specialist healthcare in Denmark, Norway, and Sweden.

### Data collection

The study was conducted in Sweden from September to December 2023, and in Norway and Denmark during April and May 2024. A convenience sampling method was used to capture the perspectives of patients and kidney professionals from all three countries. To provide context regarding the potential reach of the survey, Table 1 summarizes the approximate membership numbers of the national kidney patient, nephrology nurse, and nephrologist organizations that distributed the survey invitation in each country. These figures reflect the number of individuals who could potentially have received the survey through organizational communication channels. However, the survey link may also have been accessed by additional eligible participants outside these organizations.

**Table 1:** Survey recruitment organizations and membership statistics in Denmark, Sweden and Norway

Country	Organization	Number of members	Data collection year
Denmark	Danish Kidney Association	6,200	2024
	Danish Society of Nephrology Nurses	125	2024
	Danish Society of Nephrology	319	2024
Sweden	The Swedish Kidney Patients’ Association	4,200	2023
	Swedish Nephrology Nurses Association	252	2023
	Swedish Society of Nephrology	499	2023
Norway	The national Association for Kidney Patients and Organ Transplant Recipient	3,538	2024
	The kidney nurses’ group, the Norwegian Nurses Organisation	483	2024
	The Norwegian Society for Nephrology^a^	320	2024

^a^The organization did not share the survey in newsletters or on social media

### Development of the survey

**Fig. 1:**
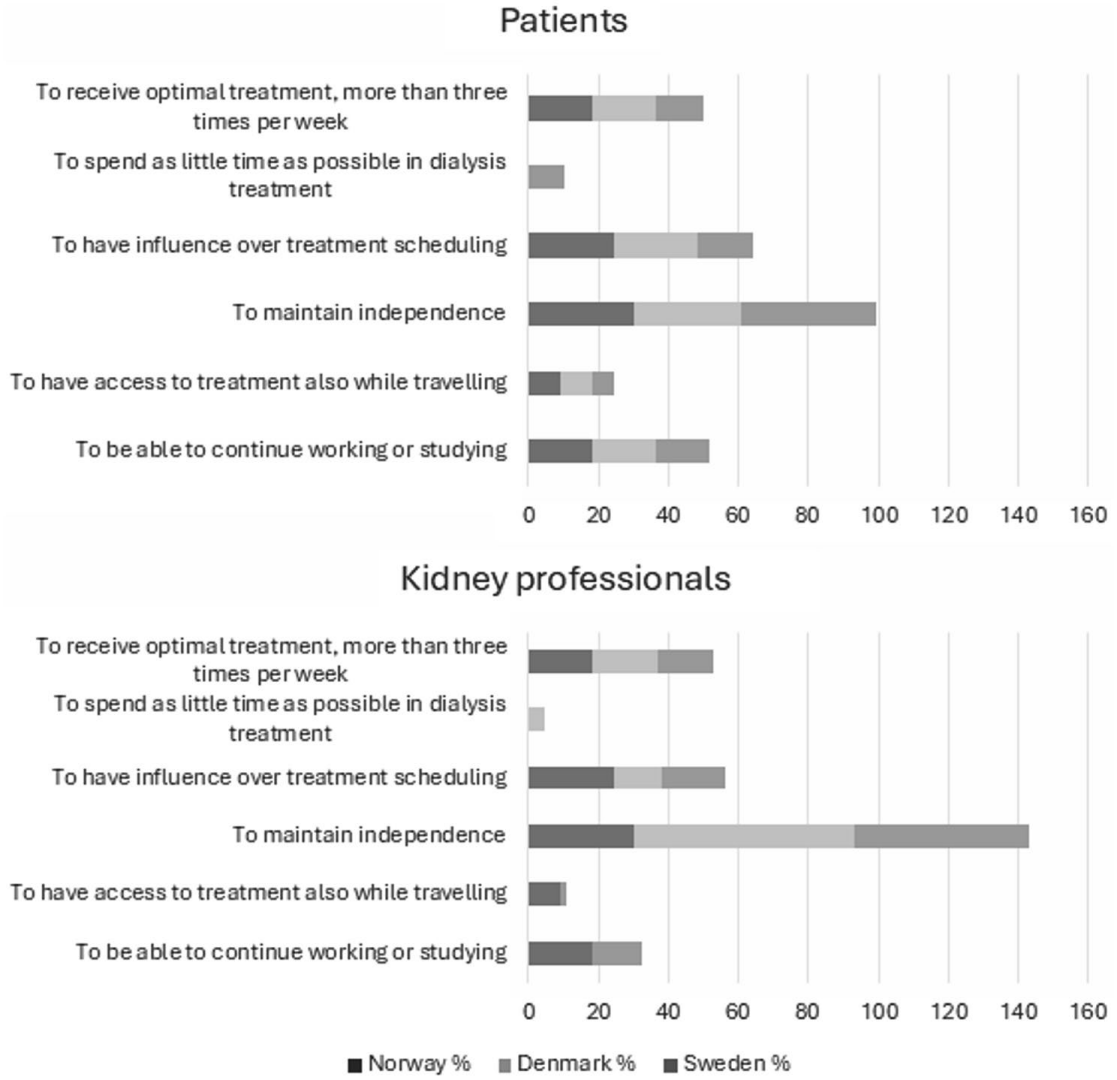
Prioritization of important factors in decision-making, stacked percentages by country - totals may exceed 100% due to multiple responses

A total of eight workshops were held in Sweden from 2021 to 2023 during the survey development. The workshops focused on the question: Why aren’t more people using home dialysis as a treatment option? During the workshops, comments were noted and organized collaboratively under different headings, such as organization, technology safety, personal conditions, etc. After each workshop, a summary was sent to the participants for possible revisions and comments. The initial workshops involved patients (n = 7), kidney professionals (n = 10) (e.g. physicians, nurses from PD, HD, and nephrology units, and dialysis technicians), and industry representatives (n = 15), followed by a combined workshop where all groups discussed the statements (n = 20). An additional two workshops were held with patients (n = 7) to finalize the survey, as well as two digital workshops with kidney failure coordinators and healthcare managers. Following these steps, a pilot version of the survey was distributed to approximately 20 members of the kidney patient association for feedback. Minor adjustments were made to the wording of certain questions to improve clarity and ensure comprehensibility across participants from diverse contexts. Additionally, the following overarching question was added: “What is most important when deciding on a dialysis modality?” and was administered as a single-choice question. The full list of response options is provided in Additional file 1 and can also be seen in Fig. 1. The survey was pilot tested in the spring of 2023 among patients with kidney failure and kidney professionals (n = 25), resulting in only minor textual corrections.

The survey consisted of a series of statements addressing barriers and was organized into thematic areas: user perspective (10 statements), technology and safety in home dialysis treatments (5 statements), training and information about treatment options (4 statements), kidney professionals’ perspectives (4 statements), and the organization of home dialysis treatments (6 statements). The participants were asked to evaluate these statements based on their personal experience and viewpoints, drawing on either current or past experiences in this field. Responses were recorded using a four-point Likert scale ranging from 1, ‘strongly agree’, to 4, ‘strongly disagree’, with an additional option “Don’t know”. The collected data were dichotomized into two categories: ‘agree’ or ‘disagree’. Responses marked as ‘I don’t know’ were excluded from the analysis. Each statement was followed by an open-ended question, allowing participants to elaborate on their responses or provide additional comments.

In the spring of 2024, Danish, Norwegian and Swedish researchers (*n* = 7) collaboratively translated the survey into Danish and Norwegian, ensuring that the phrasing in each language conveyed the same meaning across all three languages. A translation of the survey into English is provided in Additional file 1.

### Data analysis

Descriptive statistics were used to summarize demographic characteristics. Responses to Likert-scale items were dichotomized for both logistic regression and chi-square analyses. “Strongly agree” and “partly agree” were categorized as agreement, while “partly disagree” and “strongly disagree” were categorized as disagreement. Responses marked as “don’t know” or left blank were excluded from the analyses. For each survey statement, logistic regression analysis was used to examine differences in agreement between patients and kidney professionals. The dependent variable was agreement with the statement (coded as 1 = agree, 0 = disagree). Respondent group (patient vs. kidney professional) was included as the main explanatory variable, and models were adjusted for age, gender, and country.

After completing the quantitative analyses, we used a descriptive qualitative content analysis inspired by Graneheim and Lundman [15], focusing on manifest content in the open-ended responses and a descriptive presentation. Through the analysis of the open-ended responses, we sought to identify statements that could provide an understanding of the quantitative results. In line with Graneheim and Lundman [15], the process was stepwise, where we (1) familiarized ourselves with the material, (2) identified manifest units of meaning, (3) set a descriptive code, and (4) grouped the codes into categories that corresponded to the previous quantitative results. This approach made it possible to integrate qualitative and quantitative data in a way that safeguarded the participants’ voices and, at the same time, provided insight into patterns and relationships within the material.

## Results

### Participant demographics

In total, 269 patients and 185 kidney professionals responded to the survey. Of these, 67 (15.66%) participants were from Norway, 82 (23.79%) from Denmark, and 305 (60.56%) from Sweden. The mean age reported for the patients was 61.3 years, and for the kidney professionals 49.4 years. Further, 112 (42.7%) of the patients and 155 (85.6%) of the kidney professionals were women. Although both patients and kidney professionals reported experience with various modalities, 179 patients had previous experience with PD (35.8%), while 66 (13.2%) had experience with HHD. At the point of data collection, 62 patients received PD (22.1%) and 31 (11.1%) HHD. Among the kidney professionals, 164 (37.1%) had previous experience with in-hospital HD, while 207 (46.2%) reported experience with both PD and HHD. Furthermore, 143 (44.3%) were involved in home dialysis treatments. The demographics are detailed in Table 2, and the survey results are in Additional file 2.

**Table 2:** Demographics

Participants’ characteristics	All participants*				Sweden			
	Patients		Kidney professionals		Patients		Kidney professionals	
	*n*	Mean (SD)	*n*	Mean (SD)	*n*	Mean (SD)	*n*	Mean (SD)
Total	269		185		208		97	
Age (years)	261	61.26(11.83)	181	49.42(10.03)	207	63.42(10.92)	97	48.84(10.54)
	n	%	n	%				
Gender (female)	112	42.7	155	85.6	74	35.6	81	83.5
Treatment								
Experience treatment								
HD at hospital	131	26.20	164	37.10	103	26.41	90	42.25
PD	179	35.80	108	23.76	145	37.18	49	23.00
HHD	66	13.20	99	22.40	41	10.51	46	21.60
Transplanted	134	24.80	74	16.74	102	25.90	28	13.15
Current treatment								
HD at hospital	53	18.93	127	39.32	43	19.82	66	45.21
PD	62	22.14	77	23.84	47	21.66	28	19.18
HHD	31	11.07	66	20.43	17	7.83	32	21.92
Transplanted	134	47.86	53	16.41	110	50.69	20	13.70

Participants’ characteristics	Denmark				Norway			
	Patients		Kidney professionals		Patients		Kidney professionals	
	*n*	Mean (SD)	*n*	Mean (SD)	*n*	Mean (SD)	*n*	Mean (SD)
Total	25		57		36		31	
Age (years)	24	51.88(12.84)	55	50.98(9.64)	30	53.83(10.64)	29	48.41(8,90)
					n	%	n	%
Gender (female)	16	66.7	49	89.1	22	73.3	25	86.2
Treatment								
Experience treatment								
HD at hospital	16	35.56	48	30.57	12	18.46	27	36.11
PD	12	26.67	39	24.84	22	33.85	17	23.61
HHD	12	26.67	36	22.93	13	20.00	17	23.61
Transplanted	5	11.11	34	21.43	18	27.69	12	16.67
Current treatment								
HD at hospital	10	40.00	36	28.57	0		25	49.02
PD	3	12.00	36	28.57	12	31.58	13	25.49
HHD	8	32.00	27	21.43	6	15.79	7	13.73
Transplanted	4	16.00	27	21.43	20	52.63	6	11.76

### Factors influencing the choice of home dialysis

According to the quantitative findings, the most frequently reported reasons for choosing dialysis modality by both patients and kidney professionals were the desire to maintain independence, followed by the need to receive optimal treatment (Fig. 1).

In the open-ended texts, many of the comments revolve around the fact that choosing modality was a complex process, where no single reason could be identified as the most important. One of the kidney professionals stated:

*All of the above statements may be relevant. Every patient wants the treatment that is best suited for them personally*,* so what matters most will vary from individual to individual. Therefore*,* it is important to clarify in good time what each person’s needs and wishes are*,* as well as what is practically (physiologically) possible.*

Another kidney professional wrote:

*It varies greatly and depends on the individual patient. All aspects are often important*,* but there are different priorities from patient to patient – hence the different choices of dialysis.*

In the same line of argument, one patient wrote:

*There are several options here [in this survey]*,* and it should have been possible to select more than one — for example*,* work*,* studies*,* and independence.*

### Differences between patients and kidney professionals

Patients reported factors such as the lack of a clear reimbursement system (OR = 0.29, 95% CI 0.15 to 0.55), the impact of healthcare economics (OR = 0.22, 95% CI 0.10 to 0.46), and the hospital’s focus on short-term costs associated with transitioning to home dialysis rather than considering the long-term societal benefits (OR = 0.33, 95% CI 0.15 to 0.72), as influencing the choice of dialysis modality.

An example from open-ended answers from patients addressed these issues clearly:

*The only reason I’m not currently using home hemodialysis is because I’m not eligible for reimbursement of my expenses related to HHD. I also didn’t receive any compensation from the Social Insurance Agency during my previous period of HHD. I did apply*,* but according to them*,* I didn’t qualify because I didn’t meet the minimum threshold for reimbursement — since we don’t have access to municipal water at our property. So I’m not entitled to compensation for the water consumption from our private well.*

Still, the economy and lack of resources were a prominent topic from the kidney professionals’ open-ended answers:

*A shortage of nurses and poor municipal finances are often the reasons why patients do not receive home dialysis.*

*I don’t believe the healthcare trust has enough staff to ensure that everyone facing a decision about which type of dialysis treatment suits them best receives adequate training and information about the advantages and disadvantages of the different options. At the same time*,* I believe there is neither sufficient funding nor expertise to offer patients closer follow-up from either home care services or mobile dialysis nurses who could assist with challenges related to home treatment. If home dialysis patients knew that someone could visit and provide guidance and support when needed*,* I think more people would choose to manage their dialysis independently at home.*

Additionally, some technological issues were statistically significantly more pronounced among patients than among kidney professionals for the modality choice barriers. These included the lack of mature technology for home environments (OR = 0.44, 95% CI 0.25 to 0.77), the need for remote communication regarding technical and medical issues (OR 0.18, 95% CI 0.07 to 0.42), and kidney professionals’ technological knowledge (OR 0.32, 95% CI 0.15 to 0.69).

The kidney professionals were statistically significantly more concerned about the complexity of technology (OR 3.33, 95% CI 1.93 to 5.75) and, especially in HHD, that being technically complex may affect patients’ choices (OR 2.41, 95% CI 1.17 to 4.97).

Furthermore, kidney professionals were statistically significantly more likely than patients to perceive psychological and social factors as influential to their choice of treatment modality, including a preference to avoid the hospital environment (OR = 5.39, 95% CI 1.92 to 15.12), or the opposite, the value of social contact at the hospital (OR = 7.18, 95% CI 3.60 to 14.33), concerns about assuming responsibility for treatment at home (OR 7.29, 95% CI 3.59 to 14.81), the importance of involving next of kin in decision-making (OR = 7.54, 95% CI 3.44 to 16.50), and the influence of housing size (OR 3.64, 95% CI 1.52 to 8.76).

In the open-ended text, a few patients addressed that choosing hospital dialysis instead of home dialysis was to avoid bringing “the hospital home”.

*I chose to have dialysis at the hospital to feel that my home was a sanctuary from the illness. If I had received treatment at home*,* I would have felt sicker than when I have to go somewhere for treatment.*

Patients also described the routine of going to the hospital dialysis as a way of organizing a life close to what healthy individuals would do, at the same time as being adequately cared for:

*The advantage of hemodialysis was that I got to meet people*,* I got out of the house as if I were going to work*,* I was given food and drinks at the hospital*,* and I felt well cared for*,* which allowed me to relax and trust that I was getting the help I needed.*

One of the comments from the patients describes the complexities related to choosing home dialysis quite vividly, reflecting both aspects of feelings of safety and of overwhelming responsibility, the advantages of home dialysis as a treatment modality, and the need for easy communication with the kidney professionals:

*When home dialysis was first presented to me*,* I rejected it. I felt safe at the hospital. Over time*,* I became more positive as I learned about the benefits: reduced need for medication*,* more flexibility*,* avoiding a one-hour taxi ride each way*,* being able to drink a bit more*,* and eat a bit more of the ‘forbidden’ foods. But when I started at home*,* the responsibility felt overwhelming. It probably took at least six months before I started to feel like I was mastering it. It was reassuring to know I could call the dialysis unit for help over the phone*,* but often it took a long time before someone answered. I wish there were a dedicated number for HHD support. Now*,* after 1.5 years at home*,* I wouldn’t want to switch back to the hospital.*

**Fig. 2:**
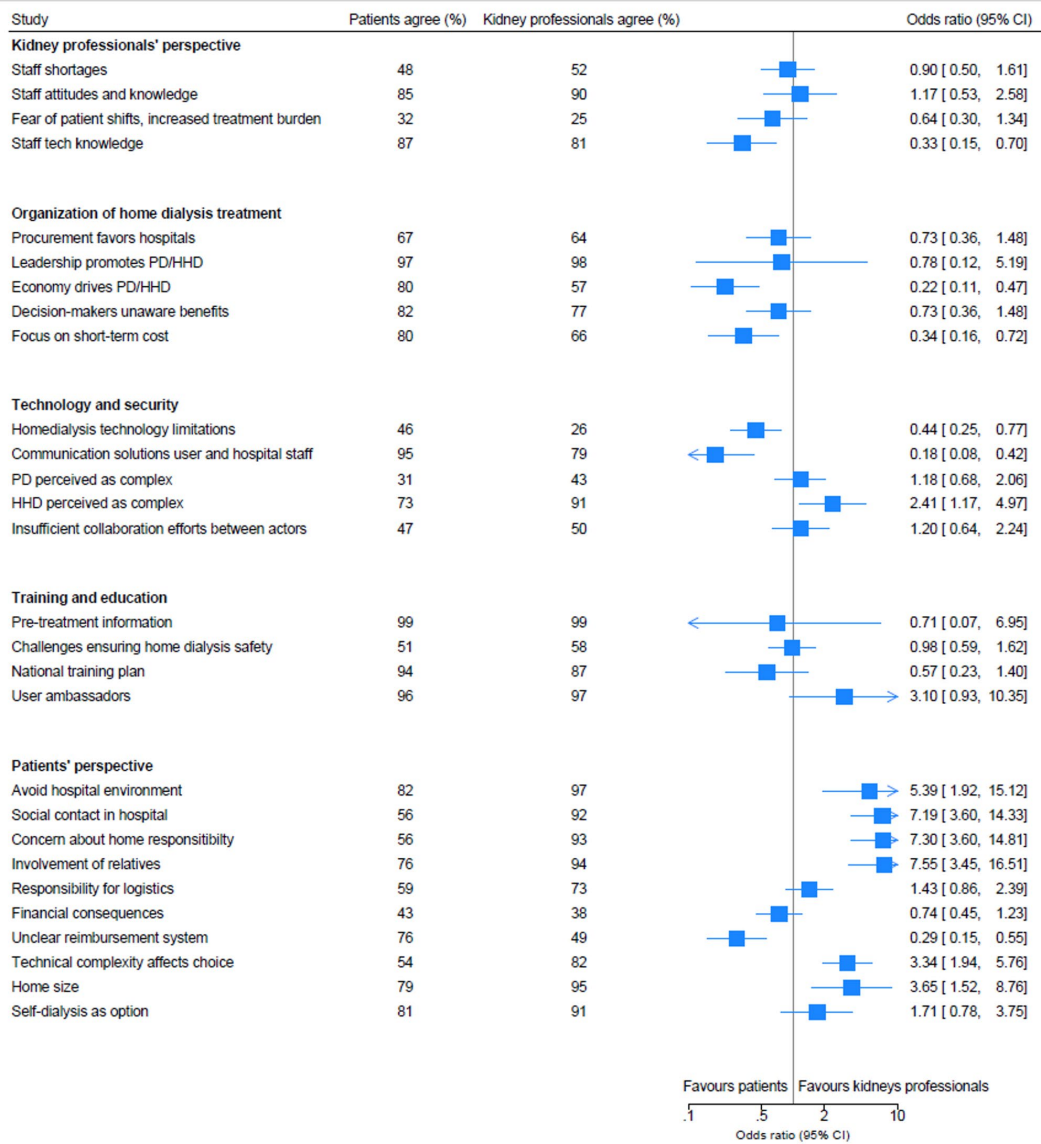
Forest plot – differences in agreement on dialysis decision-making factors: Patients versus Kidney Professionals (OR with 95% CI). Odds ratios (ORs) represent the likelihood of agreement with each statement among kidney professionals compared with patients. Models were adjusted for age, gender, and country

For further results, see Fig. 2.

### Differences between the Scandinavian countries

From the patient’s perspective, several factors influencing the choice of home dialysis differed significantly between countries. These included the perceived importance of social contact in a hospital environment, concerns about taking responsibility for treatment at home, logistics challenges, the clarity of reimbursement system, perceived technical complexity, housing conditions, and the perception that self-dialysis could be a viable option (Additional file 2).

The most significant differences between the countries were related to the importance of social contact in the hospital environment. Here, 89% (*n* = 54) of participants in Norway and 82% (*n* = 62) in Denmark agreed with the statement, compared to only 66% (*n* = 158) of participants in Sweden (*p* ≤ 0.001). Furthermore, there was a significant difference in the perceived importance of the reimbursement system, with 77% (*n* = 142) of participants in Sweden agreeing, compared to only 34% (*n* = 20) in Denmark (*p* ≤ 0.001). In Norway, there was 100% (*n* = 58) agreement that self-dialysis as an option had an impact on choice, whereas in Denmark (84%, *n* = 64) and Sweden (83%, *n* = 189), there was slightly less agreement regarding its significance (*p* = 0.003).

Only one item showed a statistically significant difference between countries regarding technological and security issues. This perception was that PD could seem complex and influence the choice, with 51% (*n* = 26) of participants agreeing in Norway, 50% (*n* = 31) in Denmark, and 30% (*n* = 71) in Sweden (*p* ≤ 0.001). Under the category of service organization, one item showed statistical differences between the countries. This item addresses the influence of economic considerations on the choice, with 48% (*n* = 19) in Norway, 65% (*n* = 39) in Denmark and 72% (*n* = 108) in Sweden (*p* = 0.014). See Additional file 2 for details.

A theme within the open-ended questions, more or less only framed among the Swedish patients, was the difference between regions concerning reimbursements. One Swedish patient wrote this:

*It’s really crazy that there’s such a big difference in*,* for example*,* compensation for HHD between the different regions.*

That there were large differences between regions in Sweden was therefore one of the significant differences between Sweden and the two other countries as well.

## Discussion

The findings of this study highlight the complex interplay of factors influencing the uptake of home dialysis in Scandinavia, underscoring both shared and divergent perceptions between patients and kidney professionals across Sweden, Norway and Denmark.

### Barriers to home dialysis uptake

The study reveals that both patients and kidney professionals recognize the multifaceted nature of choosing a dialysis modality. Independence and optimal treatment are prioritized by patients and kidney professionals in all three countries. The decision-making process is nuanced. and deeply personal as described by the patients. The qualitative insights underscore this complexity, with patients and kidney professionals emphasizing the need for individualized care plans that align with personal circumstances and preferences [16]. The variability in priorities suggests that a one-size-fits-all approach is inadequate, necessitating a person-centered care model that actively involves patients in decision-making [13].

The findings highlight that both patients and kidney professionals perceive the choice of dialysis modality not as a single decision point but as a complex and evolving process. While values such as independence and optimal treatment are central, the decision-making journey is deeply personal and unfolds over time. This underscores the importance of understanding shared decision-making and participation as ongoing processes rather than isolated events.

For patients and their significant others, this means needing continuous access to information, emotional support, and reflective dialogue throughout the decision-making trajectory. Likewise, for kidney professionals, it calls for a sustained and flexible engagement with patients, one that adapts to changing needs and priorities over time.

This process-oriented perspective on participation aligns with person-centered care models, which emphasize that genuine involvement requires more than presenting options at a single moment [17]. Instead, participation must be actively nurtured through long-term collaboration and responsiveness. Recognizing participation as a dynamic process can help build a shared understanding and foster decisions that are more aligned with the patient’s evolving context and values.

Economic and organizational barriers emerged as significant deterrents to home dialysis uptake. Patients expressed concerns about the lack of a clear reimbursement system and the healthcare system’s focus on short-term costs over long-term benefits. Furthermore, in Sweden, 77% of participants indicated that the importance of the reimbursement system played a greater role in the decision-making, a significantly higher proportion compared to Denmark and Norway. These findings suggest that financial incentives and policy reforms could play a critical role in facilitating home dialysis adoption [18]. Additionally, the shortage of nursing staff and inadequate municipal finances, as reported by kidney professionals, highlight systemic challenges that need to be addressed to support home dialysis programs effectively [18].

### Technological considerations

Technological factors also significantly impact the choice of dialysis modality [19]. Patients are particularly concerned about the maturity and availability of technology for home environments, while kidney professionals are more focused on the complexity of technology, especially for HHD. In this context, “maturity of technology” may reflect users’ perceptions of device usability, reliability in home settings, and availability of technical support or remote monitoring rather than the clinical maturity of dialysis technologies themselves. This divergence in perceptions points to the need for improved communication and education regarding technological advancements and their application in home settings. Developing user-friendly technologies and providing comprehensive training for both patients and kidney professionals could mitigate these concerns and enhance confidence in managing home dialysis [20].

### Psychological and social influences

The psychological and social dimensions of dialysis choice cannot be overlooked. This study indicates that kidney professionals perceive these factors as more influential than patients do. The preference for hospital-based dialysis to maintain social interactions and avoid bringing the “hospital home” highlights the importance of addressing emotional and social needs in dialysis care. Creating supportive networks and ensuring regular communication with kidney professionals can help alleviate feelings of isolation and responsibility associated with home dialysis. This is supported by evidence showing that stronger social support predicts greater satisfaction, enhanced quality of life, and fewer hospital admissions among dialysis patients [21, 22].

### Country-specific insights and implications

The differences observed between Scandinavian countries suggest that national healthcare policies and cultural contexts influence dialysis modality choices [5]. Sweden’s distinct stance compared to Denmark and Norway may reflect variations in healthcare delivery models and patient involvement strategies. Furthermore, the reimbursement system in Sweden is more complex due to considerable regional autonomy in healthcare administration, compared to Denmark and Norway. In Scandinavia, patients in all three countries are generally reimbursed for costs related to necessary medical equipment. However, there are important differences in whether patients are also compensated for additional household expenses associated with home dialysis, such as increased electricity and water consumption, as well as costs related to home modifications. In Sweden, reimbursement for these ancillary expenses is not standardized and may vary substantially between municipalities. Understanding these country-specific dynamics is crucial for tailoring interventions and policies that promote home dialysis [23, 24].

### Leadership and person-centered care

Leadership plays a pivotal role in advancing home dialysis [25]. The overwhelming agreement among participants on the importance of organizational support for home dialysis self-care underscores the need for strong leadership commitment. By fostering a culture of person-centered care, healthcare leaders can instill confidence in patients and kidney professionals, facilitating the transition to home dialysis [26].

### Limitations and future directions

This study has several limitations. First, convenience sampling and recruitment through newsletters, social media, and other channels of national kidney organizations may limit generalizability, and the representativeness of the sample cannot be fully determined. Because the survey was distributed through these channels, the exact number of individuals who received or viewed the invitation could not be determined, and a response rate could therefore not be calculated. Second, although the survey was developed through an extensive co-creation process involving patients and kidney professionals, it was not a standardized instrument, and its reliability was not formally tested. To enable direct comparison between patients and healthcare professionals, the same set of statements was administered to both groups. However, some items may fall outside the experiential scope of certain respondents, meaning that some responses may reflect general perceptions rather than firsthand experience. Consequently, group differences should be interpreted with caution. Third, the study collected a limited set of demographic and clinical background variables, which restricted the ability to conduct more detailed subgroup analyses. In addition, both patients and healthcare professionals may have had experience with more than one dialysis modality, making it difficult to attribute perspectives to a single treatment modality and limiting direct comparisons between modalities. Furthermore, detailed information on dialysis techniques and treatment assistance was not collected. For example, we did not distinguish between assisted and self-managed peritoneal dialysis, between different PD modalities (e.g., CAPD vs. APD), or between specific home hemodialysis regimens. These variations may influence perceived treatment complexity as well as patients’ experiences of autonomy and treatment burden. Differences in the availability and implementation of certain modalities across the participating countries, such as the limited use of assisted peritoneal dialysis in Sweden, may also affect comparability. Finally, although statistically significant differences were observed between countries, these differences primarily reflect variations in perceptions rather than large structural contrasts between the healthcare systems.

## Conclusion

This study highlights the critical need for a more systematic person-centered approach to dialysis modality choice. By addressing economic, technological, and psychosocial barriers and fostering strong leadership and patient involvement, healthcare systems can enhance the uptake of home dialysis, ultimately improving patient outcomes and quality of life.

## Supplementary Information

Below is the link to the electronic supplementary material.

Supplementary Material 1: Additional file 1: The survey (English translation).

Supplementary Material 2: Additional file 2: Survey results.

## Data Availability

The data will be shared on a reasonable request by contacting the corresponding author.

## Abbreviations

CI: Confidence Interval
HHD: Home hemodialysis
OR: Odds Ratio
PD: Peritoneal dialysis
