# Supplementary material for: Factors influencing home dialysis choice in Scandinavia: a cross-sectional study
Source: BMC Nephrol. 2026 Apr 14;27:335. doi: 10.1186/s12882-026-04972-4 (PMC13202835; doi:10.1186/s12882-026-04972-4)
Supplement: Supplementary file 1 — Supplementary Material 1: Additional file 1: The survey (English translation). [file 12882_2026_4972_MOESM1_ESM.docx]

**Additional file 1: The survey**

|  | |  |
| --- | --- | --- |
| Overall question: What is most important when deciding on a dialysis modality? | | |
|  | | To receive optimal treatment, more than three times per week |
|  | | To spend as little time as possible in dialysis treatment |
|  | | To have influence over treatment scheduling |
|  | | To maintain independence |
|  | | To have access to treatment also while travelling |
|  | | To be able to continue working or studying |
| Patient perspective | | **The following statements concern the patients' perspective** |
|  | Avoid hospital environment | The choice of treatment can be influenced by the fact that the user does not want to be in a hospital environment |
|  | Social contact | The choice to be treated in a hospital may, for some, be about social contact, which can be lacking if one receives treatment at home |
|  | Concern about home responsibility | There is concern or uncertainty about what it means to take responsibility for one's own treatment at home |
|  | Involvement of relatives | It is important that relatives are involved throughout the process when the patient is to choose a treatment option |
|  | Responsibility for logistics | The responsibility for, among other things, handling orders, storing materials, waste, and transportation in home treatment affects the choice of treatment option |
|  | Financial consequences | The choice of treatment can be influenced by the financial consequences due to increased expenses |
|  | Unclear reimbursement system | There is no clear reimbursement system for patients who choose to receive treatment at home |
|  | Technical complexity | The technical aspects can be perceived as complicated and affect the choice of treatment |
|  | Home size | The size of the home can affect the choice of treatment |
|  | Self-dialysis as option | If the necessary conditions for self-dialysis at the hospital were created, it would be a good alternative, and perhaps a step towards more HHD |
| Technology and security | | **The following statements concern technology and safety around home dialysis treatment** |
|  | Home dialysis technology limitations | The technology for hemodialysis is not sufficiently developed or adapted for the home environment, which may affect the choice of treatment |
|  | Communication solutions | A prerequisite for home treatment is that there are solutions that facilitate communication between the user at home and hospital staff, both for technical and medical advice |
|  | PD perceived as complex | Receiving PD at home can be perceived as too technically advanced and can be discouraging for some |
|  | HHD perceived as complex | Receiving HHD can be perceived as too technically advanced and can be discouraging for some |
|  | Insufficient coordination efforts | Collaboration between the various actors involved in dialysis treatment is inadequate when it comes to improving the coordination of home dialysis |
| Training and education | | **The following statements concern training and information about the different treatment options** |
|  | Pre-treatment information | Before choosing treatment, it is important that users are well informed about what the treatment involves, including impacts on life situation, medical complications, and limitations in mobility |
|  | Challenges ensuring safety | It is sometimes difficult for staff, together with the user, to create a sense of security when choosing home dialysis |
|  | National training plan | A nationally designed training plan for everyone who is about to choose a treatment to replace kidney function could help more people choose home dialysis as a treatment option |
|  | User ambassadors | Users who have already chosen home dialysis should be able to act as "ambassadors" to inform other users who are about to start dialysis |
| Kidney professionals' perspective | | **The following statements concern the kidney professional's perspective** |
|  | Staff shortages | Staff turnover and shortages result in there not being enough resources to train users in home dialysis |
|  | Staff attitudes and knowledge | Staff attitudes, knowledge, and experiences influence users’ choice of treatment |
|  | Fear of patient shifts | Among staff, there is a fear that if the number of users who perform their dialysis at home increases, only the patients with the most complex care needs will remain in the hospital |
|  | Staff tech knowledge | Staff knowledge of home dialysis technology can influence users’ choice of treatment |
| Organization of home dialysis treatment | | **The following statements concern the organization of home dialysis treatment** |
|  | Procurement favors hospitals | Tendering and procurement in the healthcare system are often based on the premise that treatment should primarily be carried out in a hospital and not at home |
|  | Leadership promotes PD/HHD | The choice of home dialysis is influenced by whether the management at the nephrology clinic actively works to increase the number of home dialysis cases |
|  | Leadership enables self-care | It is important that there is a management that gives departments the opportunity to develop self-care with the help of home dialysis |
|  | Economy drives PD/HHD | It is the economics of the healthcare system that govern the investment in home dialysis |
|  | Decision-makers unaware benefits | Decision-makers in the healthcare system are not aware of the benefits of more users having dialysis at home |
|  | Focus on short-term cost | In hospitals, more focus is placed on short-term costs that a transition would involve, instead of the long-term societal benefits |
