# Supplementary material for: Factors influencing home dialysis choice in Scandinavia: a cross-sectional study
Source: BMC Nephrol. 2026 Apr 14;27:335. doi: 10.1186/s12882-026-04972-4 (PMC13202835; doi:10.1186/s12882-026-04972-4)
Supplement: Supplementary file 2 — Supplementary Material 2: Additional file 2: Survey results. [file 12882_2026_4972_MOESM2_ESM.docx]

| **Additional file 2a** Survey results for all participants, country-specific differences, *p*-values from Chi-square tests | | | | | | | |
| --- | --- | --- | --- | --- | --- | --- | --- |
|  | |  | **All participants** | | | |  |
|  | |  | Agree | | Disagree | | *P*-value |
|  | |  | n | % | n | % |  |
| **Patient perspective** | | |  |  | | |  |
|  | Avoid hospital environment |  |  |  | | |  |
|  | | Patient | 206 | 82.07 | 45 | 17.93 |  |
|  | | HCP | 174 | 97.21 | 5 | 2.79 |  |
|  | | Total | 380 |  | 50 |  | 0.071 |
|  | Social contact |  |  |  | | |  |
|  | | Patient | 112 | 55.72 | 89 | 44.28 |  |
|  | | HCP | 162 | 92.05 | 14 | 7.95 |  |
|  | | Total | 274 |  | 103 |  | **<0.001** |
|  | Concern about home responsibility |  |  |  | | |  |
|  | | Patient | 143 | 55.64 | 114 | 44.36 |  |
|  | | HCP | 167 | 92.78 | 13 | 7.22 |  |
|  | | Total | 310 |  | 127 |  | **0.005** |
|  | Involvement of relatives |  |  |  | | |  |
|  | | Patient | 192 | 76.19 | 60 | 23.81 |  |
|  | | HCP | 169 | 94.41 | 10 | 5.59 |  |
|  | | Total | 361 |  | 70 |  | 0.572 |
|  | Responsibility for logistics |  |  |  | | |  |
|  | | Patient | 153 | 58.85 | 107 | 41.15 |  |
|  | | HCP | 123 | 72.78 | 46 | 27.22 |  |
|  | | Total | 276 |  | 153 |  | **0.034** |
|  | Financial consequences |  |  |  | | |  |
|  | | Patient | 102 | 43.04 | 135 | 56.96 |  |
|  | | HCP | 60 | 37.97 | 98 | 62.03 |  |
|  | | Total | 162 |  | 233 |  | 0.096 |
|  | Unclear reimbursement system |  |  |  | | |  |
|  | | Patient | 128 | 75.74 | 41 | 24.26 |  |
|  | | HCP | 60 | 48.78 | 63 | 51.22 |  |
|  | | Total | 188 |  | 104 |  | **<0.001** |
|  | Technical complexity |  |  |  | | |  |
|  | | Patient | 141 | 53.82 | 121 | 46.18 |  |
|  | | HCP | 145 | 82.39 | 31 | 17.61 |  |
|  | | Total | 286 |  | 152 |  | **0.003** |
|  | Home size |  |  |  | | |  |
|  | | Patient | 200 | 79.05 | 53 | 20.95 |  |
|  | | HCP | 171 | 95 | 9 | 5.00 |  |
|  | | Total | 371 |  | 62 |  | **0.015** |
|  | Self-dialysis as option |  |  |  | | |  |
|  | | Patient | 153 | 80.95 | 36 | 19.05 |  |
|  | | HCP | 158 | 91.33 | 15 | 8.67 |  |
|  | | Total | 311 |  | 51 |  | **0.003** |
| **Technology and security** | | |  |  | | |  |
|  | Home dialysis technology limitations |  |  |  | | |  |
|  | | Patient | 75 | 46.01 | 88 | 53.99 |  |
|  | | HCP | 39 | 25.66 | 113 | 74.34 |  |
|  | | Total | 114 |  | 201 |  | 0.127 |
|  | Communication solutions |  |  |  | | |  |
|  | | Patient | 228 | 95 | 12 | 5 |  |
|  | | HCP | 136 | 79.07 | 36 | 20.93 |  |
|  | | Total | 364 |  | 48 |  | 0.086 |
|  | PD perceived as complex |  |  |  | | |  |
|  | | Patient | 61 | 30.96 | 136 | 69.04 |  |
|  | | HCP | 67 | 43.23 | 88 | 56.77 |  |
|  | | Total | 128 |  | 224 |  | **<0.001** |
|  | HHD perceived as complex |  |  |  | | |  |
|  | | Patient | 141 | 73.44 | 51 | 26.56 |  |
|  | | HCP | 152 | 91.02 | 15 | 8.98 |  |
|  | | Total | 293 |  | 66 |  | 0.114 |
|  | Insufficient coordination efforts |  |  |  | | |  |
|  | | Patient | 43 | 46.74 | 49 | 53.26 |  |
|  | | HCP | 66 | 50 | 66 | 50 |  |
|  | | Total | 109 |  | 115 |  | 0.191 |
| **Training and education** | | |  |  | | |  |
|  | Pre-treatment information |  |  |  | | |  |
|  | | Patient | 252 | 99.21 | 2 | 0.79 |  |
|  | | HCP | 177 | 98.88 | 2 | 1.12 |  |
|  | | Total | 429 |  | 4 |  | 0.105 |
|  | Challenges ensuring safety |  |  |  | | |  |
|  | | Patient | 103 | 50.99 | 99 | 49.01 |  |
|  | | HCP | 96 | 58.18 | 69 | 41.82 |  |
|  | | Total | 199 |  | 168 |  | 0.525 |
|  | National training plan |  |  |  | | |  |
|  | | Patient | 174 | 93.55 | 12 | 6.45 |  |
|  | | HCP | 115 | 87.12 | 17 | 12.88 |  |
|  | | Total | 289 |  | 29 |  | 0.154 |
|  | User ambassadors |  |  |  | | |  |
|  | | Patient | 226 | 95.76 | 10 | 4.24 |  |
|  | | HCP | 170 | 97.14 | 5 | 2.86 |  |
|  | | Total | 396 |  | 15 |  | 0.330 |
| **Kidney professionals' perspective** | | |  |  | | |  |
|  | Staff shortages |  |  |  | | |  |
|  | | Patient | 59 | 48.36 | 63 | 51.64 |  |
|  | | HCP | 88 | 52.07 | 81 | 47.93 |  |
|  | | Total | 147 |  | 144 |  | 0.073 |
|  | Staff attitudes and knowledge |  |  |  | | |  |
|  | | Patient | 175 | 84.95 | 31 | 15.05 |  |
|  | | HCP | 158 | 90.29 | 17 | 9.71 |  |
|  | | Total | 333 |  | 48 |  | **0.012** |
|  | Fear of patient shifts |  |  |  | | |  |
|  | | Patient | 25 | 32.05 | 53 | 67.95 |  |
|  | | HCP | 40 | 24.84 | 121 | 75.16 |  |
|  | | Total | 65 |  | 174 |  | 0.470 |
|  | Staff tech knowledge |  |  |  | | |  |
|  | | Patient | 162 | 86.63 | 25 | 13.37 |  |
|  | | HCP | 133 | 80.61 | 32 | 19.39 |  |
|  | | Total | 295 |  | 57 |  | 0.870 |
| **Organisation of home dialysis treatment** | | |  |  | | |  |
|  | Procurement favours hospitals | |  |  | | |  |
|  | | Patient | 56 | 66.67 | 28 | 33.33 |  |
|  | | HCP | 76 | 63.87 | 43 | 36.13 |  |
|  | | Total | 132 |  | 71 |  | 0.230 |
|  | Leadership promotes PD/HHD | |  |  | | |  |
|  | | Patient | 146 | 97.33 | 4 | 2.67 |  |
|  | | HCP | 171 | 97.71 | 4 | 2.29 |  |
|  | | Total | 317 |  | 8 |  | 0.280 |
|  | Leadership enables self-care | |  |  | | |  |
|  | | Patient | 195 | 99.49 | 1 | 0.51 |  |
|  | | HCP | 179 | 100 | 0 | 0.00 |  |
|  | | Total | 374 |  | 1 |  | 0.760 |
|  | Economy drives PD/HHD | |  |  | | |  |
|  | | Patient | 82 | 80.39 | 20 | 19.61 |  |
|  | | HCP | 84 | 56.76 | 64 | 43.24 |  |
|  | | Total | 166 |  | 84 |  | **0.014** |
|  | Decision-makers unaware benefits | |  |  | | |  |
|  | | Patient | 95 | 81.9 | 21 | 18.1 |  |
|  | | HCP | 114 | 76.51 | 35 | 23.49 |  |
|  | | Total | 209 |  | 56 |  | 0.101 |
|  | Focus on short-term cost | |  |  | | |  |
|  | | Patient | 74 | 79.57 | 19 | 20.43 |  |
|  | | HCP | 97 | 66.44 | 49 | 33.56 |  |
|  | | Total | 171 |  | 68 |  | 0.168 |
| Note: The total number of respondents was 260 patients and 185 healthcare professionals (HCPs). Not all respondents responded to every statement; therefore, denominators may vary across items. Percentages shown in the table represent the distribution among those who agreed or disagreed.  *P*-value refers to a significant difference in the score between the three countries  Abbreviations: HCP, Health Care Professionals; PD, Peritoneal Dialysis; HHD, Home Hemodialysis | | | | | | | |

| **Additional file 2b** Survey results Sweden | | |  |  | | |
| --- | --- | --- | --- | --- | --- | --- |
|  | |  | **Sweden** | | | |
|  | |  | Agree | | Disagree | |
|  | |  | n | % | n | % |
| **Patient perspective** | | |  |  | | |
|  | Avoid hospital environment |  |  |  | | |
|  | | Patient | 157 | 80.93 | 37 | 19.07 |
|  | | HCP | 89 | 96.74 | 3 | 3.26 |
|  | Social contact |  |  |  | | |
|  | | Patient | 73 | 48.99 | 76 | 51.01 |
|  | | HCP | 85 | 93.41 | 6 | 6.59 |
|  | Concern about home responsibility |  |  |  | | |
|  | | Patient | 105 | 53.03 | 93 | 46.97 |
|  | | HCP | 87 | 94.57 | 5 | 5.43 |
|  | Involvement of relatives |  |  |  | | |
|  | | Patient | 152 | 78.35 | 42 | 21.65 |
|  | | HCP | 84 | 92.31 | 7 | 7.69 |
|  | Responsibility for logistics |  |  |  | | |
|  | | Patient | 112 | 56.28 | 87 | 43.72 |
|  | | HCP | 61 | 70.11 | 26 | 29.89 |
|  | Financial consequences |  |  |  | | |
|  | | Patient | 74 | 41.11 | 106 | 58.89 |
|  | | HCP | 42 | 51.85 | 39 | 48.15 |
|  | Unclear reimbursement system |  |  |  | | |
|  | | Patient | 97 | 78.23 | 27 | 21.77 |
|  | | HCP | 45 | 75.00 | 15 | 25.00 |
|  | Technical complexity |  |  |  | | |
|  | | Patient | 107 | 52.45 | 97 | 47.55 |
|  | | HCP | 69 | 77.53 | 20 | 22.47 |
|  | Home size |  |  |  | | |
|  | | Patient | 148 | 76.29 | 46 | 23.71 |
|  | | HCP | 88 | 94.62 | 5 | 5.38 |
|  | Self-dialysis as option |  |  |  | | |
|  | | Patient | 105 | 76.64 | 32 | 23.36 |
|  | | HCP | 84 | 92.31 | 7 | 7.69 |
| **Technology and security** | | |  |  | | |
|  | Home dialysis technology limitations |  |  |  | | |
|  | | Patient | 53 | 44.54 | 66 | 55.46 |
|  | | HCP | 20 | 22.47 | 69 | 77.53 |
|  | Communication solutions |  |  |  | | |
|  | | Patient | 173 | 94.54 | 10 | 5.46 |
|  | | HCP | 75 | 80.65 | 18 | 19.35 |
|  | PD perceived as complex |  |  |  | | |
|  | | Patient | 47 | 29.75 | 111 | 70.25 |
|  | | HCP | 24 | 29.63 | 57 | 70.37 |
|  | HHD perceived as complex |  |  |  | | |
|  | | Patient | 103 | 70.55 | 43 | 29.45 |
|  | | HCP | 85 | 91.30 | 8 | 8.70 |
|  | Insufficient coordination efforts |  |  |  | | |
|  | | Patient | 28 | 53.85 | 24 | 46.15 |
|  | | HCP | 32 | 55.17 | 26 | 44.83 |
| **Training and education** | | |  |  | | |
|  | Pre-treatment information |  |  |  | | |
|  | | Patient | 196 | 99.49 | 1 | 0.51 |
|  | | HCP | 93 | 98.94 | 1 | 1.06 |
|  | Challenges ensuring safety |  |  |  | | |
|  | | Patient | 77 | 51.33 | 73 | 48.67 |
|  | | HCP | 48 | 56.47 | 37 | 43.53 |
|  | National training plan |  |  |  | | |
|  | | Patient | 134 | 93.71 | 9 | 6.29 |
|  | | HCP | 68 | 90.67 | 7 | 9.33 |
|  | User ambassadors |  |  |  | | |
|  | | Patient | 174 | 96.13 | 7 | 3.87 |
|  | | HCP | 92 | 98.92 | 1 | 1.08 |
| **Kidney professionals' perspective** | | |  |  | | |
|  | Staff shortages |  |  |  | | |
|  | | Patient | 39 | 45.88 | 46 | 54.12 |
|  | | HCP | 51 | 53.68 | 44 | 46.32 |
|  | Staff attitudes and knowledge |  |  |  | | |
|  | | Patient | 129 | 83.23 | 26 | 16.77 |
|  | | HCP | 78 | 84.78 \| | 14 | 15.22 |
|  | Fear of patient shifts |  |  |  | | |
|  | | Patient | 12 | 24.00 | 38 | 76.00 |
|  | | HCP | 22 | 25.88 | 63 | 74.12 |
|  | Staff tech knowledge |  |  |  | | |
|  | | Patient | 118 | 85.51 | 20 | 14.49 |
|  | | HCP | 73 | 79.35 | 19 | 20.65 |
| **Organisation of home dialysis treatment** | | |  |  | | |
|  | Procurement favours hospitals | |  |  | | |
|  | | Patient | 40 | 65.57 | 21 | 34.43 |
|  | | HCP | 48 | 68.57 | 22 | 31.43 |
|  | Leadership promotes PD/HHD | |  |  | | |
|  | | Patient | 103 | 96.26 | 4 | 3.74 |
|  | | HCP | 91 | 96.81 | 3 | 3.19 |
|  | Leadership enables self-care | |  |  | | |
|  | | Patient | 146 | 99.32 | 1 | 0.68 |
|  | | HCP | 95 | 100.00 | 0 | 0.00 |
|  | Economy drives PD/HHD | |  |  | | |
|  | | Patient | 61 | 85.92 | 10 | 14.08 |
|  | | HCP | 47 | 59.49 | 32 | 40.51 |
|  | Decision-makers unaware benefits | |  |  | | |
|  | | Patient | 67 | 82.72 | 14 | 17.28 |
|  | | HCP | 65 | 81.25 | 15 | 18.75 |
|  | Focus on short-term cost | |  |  | | |
|  | | Patient | 53 | 84.13 | 10 | 15.87 |
|  | | HCP | 50 | 68.49 | 23 | 31.51 |
| Note: The total number of respondents was 208 patients and 97 healthcare professionals (HCPs). Not all respondents responded to every statement; therefore, denominators may vary across items. Percentages shown in the table represent the distribution among those who agreed or disagreed.  Abbreviations: HCP, Health Care Professionals; PD, Peritoneal Dialysis; HHD, Home Hemodialysis | | | | | | |

| **Additional file 2c** Survey results Denmark | | |  |  | | |
| --- | --- | --- | --- | --- | --- | --- |
|  | |  | **Denmark** | | | |
|  | |  | Agree | | Disagree | |
|  | |  | n | % | n | % |
| **Patient perspective** | | |  |  | | |
|  | Avoid hospital environment |  |  |  | | |
|  | | Patient | 20 | 86.96 | 3 | 13.04 |
|  | | HCP | 56 | 98.25 | 1 | 1.75 |
|  | Social contact |  |  |  | | |
|  | | Patient | 15 | 68.18 | 7 | 31.82 |
|  | | HCP | 47 | 87.04 | 7 | 12.96 |
|  | Concern about home responsibility |  |  |  | | |
|  | | Patient | 13 | 54.17 | 11 | 45.83 |
|  | | HCP | 49 | 85.96 | 8 | 14.04 |
|  | Involvement of relatives |  |  |  | | |
|  | | Patient | 17 | 70.83 | 7 | 29.17 |
|  | | HCP | 54 | 94.74 | 3 | 5.26 |
|  | Responsibility for logistics |  |  |  | | |
|  | | Patient | 17 | 68.00 | 8 | 32.00 |
|  | | HCP | 36 | 67.92 | 17 | 32.08 |
|  | Financial consequences |  |  |  | | |
|  | | Patient | 14 | 56.00 | 11 | 44.00 |
|  | | HCP | 9 | 18.00 | 41 | 82.00 |
|  | Unclear reimbursement system |  |  |  | | |
|  | | Patient | 10 | 62.50 | 6 | 37.50 |
|  | | HCP | 10 | 23.26 | 33 | 76.74 |
|  | Technical complexity |  |  |  | | |
|  | | Patient | 10 | 50.00 | 10 | 50.00 |
|  | | HCP | 46 | 82.14 | 10 | 17.86 |
|  | Home size |  |  |  | | |
|  | | Patient | 21 | 91.30 | 2 | 8.70 |
|  | | HCP | 53 | 94.64 | 3 | 5.36 |
|  | Self-dialysis as option |  |  |  | | |
|  | | Patient | 19 | 82.61 | 4 | 17.39 |
|  | | HCP | 45 | 84.91 | 8 | 15.09 |
| **Technology and security** | | |  |  | | |
|  | Home dialysis technology limitations |  |  |  | | |
|  | | Patient | 9 | 50.00 | 9 | 50.00 |
|  | | HCP | 8 | 20.51 | 31 | 79.49 |
|  | Communication solutions |  |  |  | | |
|  | | Patient | 23 | 100.00 | 0 | 0.00 |
|  | | HCP | 36 | 72.00 | 14 | 28.00 |
|  | PD perceived as complex |  |  |  | | |
|  | | Patient | 2 | 14.29 | 12 | 85.71 |
|  | | HCP | 29 | 60.42 | 19 | 39.58 |
|  | HHD perceived as complex |  |  |  | | |
|  | | Patient | 16 | 80.00 | 4 | 20.00 |
|  | | HCP | 44 | 89.80 | 5 | 10.20 |
|  | Insufficient coordination efforts |  |  |  | | |
|  | | Patient | 9 | 60.00 | 6 | 40.00 |
|  | | HCP | 20 | 40.82 | 29 | 59.18 |
| **Training and education** | | |  |  | | |
|  | Pre-treatment information |  |  |  | | |
|  | | Patient | 25 | 100.00 | 0 | 0.00 |
|  | | HCP | 55 | 100.00 | 0 | 0.00 |
|  | Challenges ensuring safety |  |  |  | | |
|  | | Patient | 15 | 62.50 | 9 | 37.50 |
|  | | HCP | 31 | 58.49 | 22 | 41.51 |
|  | National training plan |  |  |  | | |
|  | | Patient | 17 | 89.47 | 2 | 10.53 |
|  | | HCP | 25 | 80.65 | 6 | 19.35 |
|  | User ambassadors |  |  |  | | |
|  | | Patient | 23 | 95.83 | 1 | 4.17 |
|  | | HCP | 49 | 92.45 | 4 | 7.55 |
| **Kidney professionals' perspective** | | |  |  | | |
|  | Staff shortages |  |  |  | | |
|  | | Patient | 9 | 52.94 | 8 | 47.06 |
|  | | HCP | 19 | 38.78 | 30 | 61.22 |
|  | Staff attitudes and knowledge |  |  |  | | |
|  | | Patient | 21 | 95.45 | 1 | 4.55 |
|  | | HCP | 52 | 96.30 | 2 | 3.70 |
|  | Fear of patient shifts |  |  |  | | |
|  | | Patient | 8 | 80.00 | 2 | 20.00 |
|  | | HCP | 12 | 24.00 | 38 | 76.00 |
|  | Staff tech knowledge |  |  |  | | |
|  | | Patient | 19 | 86.36 | 3 | 13.64 |
|  | | HCP | 38 | 84.44 | 7 | 15.56 |
| **Organisation of home dialysis treatment** | | |  |  | | |
|  | Procurement favours hospitals | |  |  | | |
|  | | Patient | 9 | 90.00 | 1 | 10.00 |
|  | | HCP | 19 | 61.29 | 12 | 38.71 |
|  | Leadership promotes PD/HHD | |  |  | | |
|  | | Patient | 15 | 100.00 | 0 | 0.00 |
|  | | HCP | 52 | 98.11 | 1 | 1.89 |
|  | Leadership enables self-care | |  |  | | |
|  | | Patient | 20 | 100.00 | 0 | 0.00 |
|  | | HCP | 55 | 100.00 | 0 | 0.00 |
|  | Economy drives PD/HHD | |  |  | | |
|  | | Patient | 13 | 86.67 | 2 | 13.33 |
|  | | HCP | 26 | 57.78 | 19 | 42.22 |
|  | Decision-makers unaware benefits | |  |  | | |
|  | | Patient | 13 | 86.67 | 2 | 13.33 |
|  | | HCP | 33 | 76.74 | 10 | 23.26 |
|  | Focus on short-term cost | |  |  | | |
|  | | Patient | 11 | 91.67 | 1 | 8.33 |
|  | | HCP | 32 | 64.00 | 18 | 36.00 |
| Note: The total number of respondents was 25 patients and 57 healthcare professionals (HCPs). Not all respondents responded to every statement; therefore, denominators may vary across items. Percentages shown in the table represent the distribution among those who agreed or disagreed.  Abbreviations: HCP, Health Care Professionals; PD, Peritoneal Dialysis; HHD, Home Hemodialysis | | | | | | |

| **Additional file 2d** Survey results Norway | | |  |  | | |
| --- | --- | --- | --- | --- | --- | --- |
|  | |  | **Norway** | | | |
|  | |  | Agree | | Disagree | |
|  | |  | n | % | n | % |
| **Patient perspective** | | |  |  | | |
|  | Avoid hospital environment |  |  |  | | |
|  | | Patient | 29 | 85.29 | 5 | 14.71 |
|  | | HCP | 29 | 96.67 | 1 | 3.33 |
|  | Social contact |  |  |  | | |
|  | | Patient | 24 | 80.00 | 6 | 20.00 |
|  | | HCP | 30 | 96.77 | 1 | 3.23 |
|  | Concern about home responsibility |  |  |  | | |
|  | | Patient | 25 | 71.43 | 10 | 28.57 |
|  | | HCP | 31 | 100.00 | 0 | 0.00 |
|  | Involvement of relatives |  |  |  | | |
|  | | Patient | 23 | 67.65 | 11 | 32.35 |
|  | | HCP | 31 | 100.00 | 0 | 0.00 |
|  | Responsibility for logistics |  |  |  | | |
|  | | Patient | 24 | 66.67 | 12 | 33.33 |
|  | | HCP | 26 | 89.66 | 3 | 10.34 |
|  | Financial consequences |  |  |  | | |
|  | | Patient | 14 | 43.75 | 18 | 56.25 |
|  | | HCP | 9 | 33.33 | 18 | 66.67 |
|  | Unclear reimbursement system |  |  |  | | |
|  | | Patient | 21 | 72.41 | 8 | 27.59 |
|  | | HCP | 5 | 25.00 | 15 | 75.00 |
|  | Technical complexity |  |  |  | | |
|  | | Patient | 23 | 63.89 | 13 | 36.11 |
|  | | HCP | 30 | 96.77 | 1 | 3.23 |
|  | Home size |  |  |  | | |
|  | | Patient | 31 | 86.11 | 5 | 13.89 |
|  | | HCP | 30 | 96.77 | 1 | 3.23 |
|  | Self-dialysis as option |  |  |  | | |
|  | | Patient | 29 | 100.00 | 0 | 0.00 |
|  | | HCP | 29 | 100.00 | 0 | 0.00 |
| **Technology and security** | | |  |  | | |
|  | Home dialysis technology limitations |  |  |  | | |
|  | | Patient | 13 | 50.00 | 13 | 50.00 |
|  | | HCP | 11 | 45.83 | 13 | 54.17 |
|  | Communication solutions |  |  |  | | |
|  | | Patient | 32 | 94.12 | 2 | 5.88 |
|  | | HCP | 25 | 86.21 | 4 | 13.79 |
|  | PD perceived as complex |  |  |  | | |
|  | | Patient | 12 | 48.00 | 13 | 52.00 |
|  | | HCP | 14 | 53.85 | 12 | 46.15 |
|  | HHD perceived as complex |  |  |  | | |
|  | | Patient | 22 | 84.62 | 4 | 15.38 |
|  | | HCP | 24 | 92.31 | 2 | 7.69 |
|  | Insufficient coordination efforts |  |  |  | | |
|  | | Patient | 6 | 24.00 | 19 | 76.00 |
|  | | HCP | 14 | 56.00 | 11 | 44.00 |
| **Training and education** | | |  |  | | |
|  | Pre-treatment information |  |  |  | | |
|  | | Patient | 31 | 96.88 | 1 | 3.12 |
|  | | HCP | 29 | 96.67 | 1 | 3.33 |
|  | Challenges ensuring safety |  |  |  | | |
|  | | Patient | 11 | 39.29 | 17 | 60.71 |
|  | | HCP | 17 | 62.96 | 10 | 37.04 |
|  | National training plan |  |  |  | | |
|  | | Patient | 23 | 95.83 | 1 | 4.17 |
|  | | HCP | 22 | 84.62 | 4 | 15.38 |
|  | User ambassadors |  |  |  | | |
|  | | Patient | 29 | 93.55 | 2 | 6.45 |
|  | | HCP | 29 | 100.00 | 0 | 0.00 |
| **Kidney professionals' perspective** | | |  |  | | |
|  | Staff shortages |  |  |  | | |
|  | | Patient | 11 | 55.00 | 9 | 45.00 |
|  | | HCP | 18 | 72.00 | 7 | 28.00 |
|  | Staff attitudes and knowledge |  |  |  | | |
|  | | Patient | 25 | 86.21 | 4 | 13.79 |
|  | | HCP | 28 | 96.55 | 1 | 3.45 |
|  | Fear of patient shifts |  |  |  | | |
|  | | Patient | 5 | 27.78 | 13 | 72.22 |
|  | | HCP | 6 | 23.08 | 20 | 76.92 |
|  | Staff tech knowledge |  |  |  | | |
|  | | Patient | 25 | 92.59 | 2 | 7.41 |
|  | | HCP | 22 | 78.57 | 6 | 21.43 |
| **Organisation of home dialysis treatment** | | |  |  | | |
|  | Procurement favours hospitals | |  |  | | |
|  | | Patient | 7 | 53.85 | 6 | 46.15 |
|  | | HCP | 9 | 50.00 | 9 | 50.00 |
|  | Leadership promotes PD/HHD | |  |  | | |
|  | | Patient | 28 | 100.00 | 0 | 0.00 |
|  | | HCP | 28 | 100.00 | 0 | 0.00 |
|  | Leadership enables self-care | |  |  | | |
|  | | Patient | 29 | 100.00 | 0 | 0.00 |
|  | | HCP | 29 | 100.00 | 0 | 0.00 |
|  | Economy drives PD/HHD | |  |  | | |
|  | | Patient | 8 | 50.00 | 8 | 50.00 |
|  | | HCP | 11 | 45.83 | 13 | 54.17 |
|  | Decision-makers unaware benefits | |  |  | | |
|  | | Patient | 15 | 75.00 | 5 | 25.00 |
|  | | HCP | 16 | 61.54 | 10 | 38.46 |
|  | Focus on short-term cost | |  |  | | |
|  | | Patient | 10 | 55.56 | 8 | 44.44 |
|  | | HCP | 15 | 65.22 | 8 | 34.78 |
| Note: The total number of respondents was 36 patients and 31 healthcare professionals (HCPs). Not all respondents responded to every statement; therefore, denominators may vary across items. Percentages shown in the table represent the distribution among those who agreed or disagreed.  Abbreviations: HCP, Health Care Professionals; PD, Peritoneal Dialysis; HHD, Home Hemodialysis | | | | | | |
